# Vaginal vault dehiscence with small bowel evisceration, bowel necrosis, and intra-abdominal haemorrhage: a case report

**DOI:** 10.1093/jscr/rjae191

**Published:** 2024-04-01

**Authors:** Ee Thong Lim, Nicholas Stylianides, Laurentiu Craciunas, Nikolaos Tsampras

**Affiliations:** Department of Gynaecology, Manchester University NHS Foundation Trust, Oxford Rd, Manchester M13 9WL, United Kingdom; Department of General Surgery, Manchester University NHS Foundation Trust, Oxford Rd, Manchester M13 9WL, United Kingdom; Centre for Life, Newcastle Fertility Centre, Biomedicine West Wing, International Centre for Life, Times Square, Newcastle upon Tyne NE1 4EP, United Kingdom; Developmental Biology and Medicine, School of Medical Sciences, The University of Manchester, Oxford Road, Manchester M13 9PL, United Kingdom

**Keywords:** vaginal vault dehiscence, evisceration, hysterectomy

## Abstract

Vaginal vault dehiscence with evisceration is a rare but a potentially life-threatening complication of total hysterectomy that requires prompt recognition, diagnosis, and management. The overall incidence of vaginal vault dehiscence is 0.53%. The mortality rate increases to 5.6% when bowel evisceration is present. We report a case of vaginal vault dehiscence with small bowel evisceration complicated by bowel necrosis and intra-abdominal haemorrhage in a 48-year-old woman following her first sexual intercourse 4 months after her total abdominal hysterectomy.

## Introduction

Vaginal vault dehiscence is a rare but a potentially life-threatening complication of total hysterectomy. It involves partial or complete separation of the anterior and posterior edges of the vaginal cuff, which could lead to expulsion of intraperitoneal contents, with distal ileum being the most frequently eviscerating organ. The overall incidence of vaginal vault dehiscence is 0.53% [1]. Laparoscopic hysterectomies are shown to have 3-to-13-fold higher risk of vaginal cuff dehiscence when compared with open approach [1]. The mortality rate increases to 5.6% when bowel evisceration is present, because of risk of sepsis and bowel ischemia secondary to obstructed blood flow [2]. Vaginal vault dehiscence with evisceration is a surgical emergency that requires prompt diagnosis and management.

We report a case of vaginal vault dehiscence with small bowel evisceration complicated by bowel necrosis and intra-abdominal haemorrhage. We discuss this uncommon complication with potentially high mortality, highlighting its risk factors and possible steps to prevent its occurrence.

## Case presentation

A 48-year-old parity 2 (one vaginal delivery and one caesarean section) pre-menopausal woman with BMI of 32 underwent a total abdominal hysterectomy for large uterine fibroids and menorrhagia. She had a history of anterior and posterior vaginal wall prolapse and no other significant medical or family history. Her recovery was uneventful. The vaginal vault appeared well-healed at 6-week follow-up. The patient presented 3 months post-hysterectomy with acute severe abdominal pain and bowel prolapse through the introitus, following her first sexual intercourse since her hysterectomy. She felt vaginal prolapse after micturition. She reported presyncope and collapsed after calling for help.

**Figure 1:**
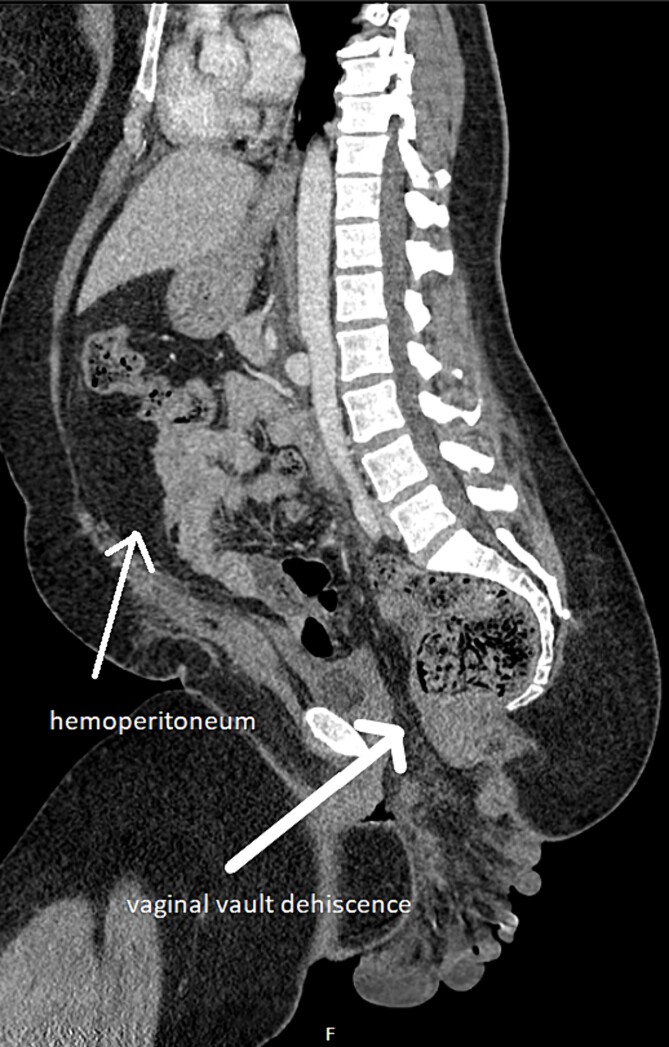
Small bowel evisceration through vaginal vault dehiscence and haemoperitoneum on CT.

On examination, she was haemodynamically unstable with signs of acute abdomen and dusky-looking bowel protruding through her vaginal vault. Computed tomography (CT) showed small bowel evisceration through the vagina (Fig. 1). Multiple loops were seen outside the pelvic floor, with poor degree of perfusion and submucosal oedema. Lobulated hyperdensity tracking around small bowel loops suggested intra-abdominal haemorrhage.

During emergency laparotomy a large volume haemoperitoneum, because of extensive shearing of the small bowel mesentery was revealed. The protruded small bowel appeared necrosed and was resected with stapled anastomosis from jejunum to terminal ileum. A segment of 220 cm from duodenojejunal flexure and 10 cm of the terminal ileum from ileocecal valve were preserved. The vaginal wall defect was closed with continuous vicryl suture.

The patient recovered well and was discharged on the 7th post-operative day. She had a follow-up 4 months after and healthy vaginal vault was noted with no palpable defect. However, there was significant negative impact on her mental and psychosexual health, with the fear to have sexual intercourse. She benefited from counselling sessions and psychosexual rehabilitation.

## Discussion

In pre-menopausal women, vaginal vault dehiscence with evisceration is rare and mostly associated with vaginal trauma from early coitus, forceful sexual practice, and instrumentation with foreign objects [2–4]. Sexual intercourse is considered the main trigger event [2, 3]. There is no current evidence to recommend the optimal time to resume sexual intercourse post-hysterectomy, although most cases occur within 4 months [3]. Poor wound healing is associated with increased risk of vaginal vault dehiscence and evisceration. This includes post-operative infection, haematoma, chronic medical illness, adjuvant chemotherapy or radiotherapy, pelvic floor weakness, and smoking [3, 4]. Electrosurgery is also a risk factor because of avascularisation of the vaginal edges [3].

A randomized trial showed that laparoscopic closure of the vaginal cuff is associated with a significant decrease in vaginal vault dehiscence, formation of cuff haematoma, and cuff infection comparing to transvaginal cuff closure [5]. The use of barbed sutures in cuff closure is also shown to reduce the risk of vaginal vault dehiscence [1]. There is a potential benefit of applying vaginal oestrogen cream for 6 weeks before hysterectomy [3].

In the present case, the patient had underlying pelvic floor weakness and initiated her first sexual intercourse 3 months post-hysterectomy that precipitated the vaginal vault dehiscence.

## Conclusion

Vaginal vault dehiscence with evisceration is a rare but potentially life-threatening complication. It requires prompt recognition and immediate surgical repair. Modifiable risk factors should be addressed prior to hysterectomy, to minimize the risks of vaginal vault dehiscence.
